# Experimental L-Band Airborne SAR for Oil Spill Response at Sea and in Coastal Waters

**DOI:** 10.3390/s18020641

**Published:** 2018-02-22

**Authors:** Cathleen E. Jones, Benjamin Holt

**Affiliations:** Jet Propulsion Laboratory, California Institute of Technology, 4800 Oak Grove Dr., Pasadena, CA 91109, USA, benjamin.holt@jpl.nasa.gov

**Keywords:** oil slicks, slick characterization, synthetic aperture radar, UAVSAR

## Abstract

Satellite synthetic aperture radar (SAR) is frequently used during oil spill response efforts to identify oil slick extent, but suffers from the major disadvantages of potential long latency between when a spill occurs and when a satellite can image the site and an inability to continuously track the spill as it develops. We show using data acquired with the Uninhabited Aerial Vehicle SAR (UAVSAR) instrument how a low noise, high resolution, L-band SAR could be used for oil spill response, with specific examples of tracking slick extent, position and weathering; determining zones of relatively thicker or more emulsified oil within a slick; and identifying oil slicks in coastal areas where look-alikes such as calm waters or biogenic slicks can confound the identification of mineral oil spills. From these key points, the essential features of an airborne SAR system for operational oil spill response are described, and further research needed to determine SAR’s capabilities and limitations in quantifying slick thickness is discussed.

## 1. Introduction

Oil spills occur throughout the world, on land and at sea, through release from pipelines, wells, transport vehicles, storage tanks and natural seeps. The larger spills most frequently occur in coastal waters of oil-producing regions. For example, the United States (U.S.) National Oceanic and Atmospheric Administration (NOAA) Office of Response and Restoration responded to 117 major spills in 2014, 88% of which occurred within U.S. borders and 38% of which occurred in the states bordering the Gulf of Mexico [1]. Major spills of crude oil from oil platforms, pipelines and tankers often occur in the open sea where the released material can be transported far from the source by winds and currents [2,3,4]. In many cases, oil reaches coastal areas, where it can adversely impact the terrestrial environment [5,6] and lead to increased erosion in marshes [7]. In fact, some forms of weathered oil can persist for years [8,9].

Minimizing the environmental impact of a spill at sea requires rapid action to stop the release and prevent the oil from spreading. Initially, responders need to know its source, the spill extent, the estimate of the quantity, the range of probable transport paths and current and future meteorological and sea conditions. Following this, responders focus on containment, recovery and clean-up activities in areas where the oil is likely to have the most severe impact. For this, they require knowledge of the thickness, volume or volumetric oil fraction to target the thickest or highest volume of oil, or areas where the oil is likely to concentrate and form persistent substances (weathered emulsions) or move into sensitive environments (coastline, nesting or spawning areas, etc.). Quantitative information about the thickness or volume is also required for some spill remediation technologies such as skimmers and dispersants.

Aerial reconnaissance to visually characterize the slick is commonly used to outline the spatial extent of the slick, estimate the rate of release and characterize the slick into known categories (e.g., silver sheen). This reconnaissance is used to separate the slick into regions that fall within standard categories that each correspond to a range of thickness, in accordance with the Bonn Agreement [10]. Visual classification can be imprecise and inconsistent; is dependent on solar illumination, viewing angle and sea state, observer training and experience; and at best determines thickness to within an order of magnitude. Remote sensing instruments are also used during response, most commonly optical or infrared sensors, side look airborne radar (SLAR) or satellite synthetic aperture radar (SAR) [11,12].

Satellite-based observations have the advantage of reliable and known orbits and, in some cases, open access data, but are not optimal for oil spill response. Although satellite-borne SARs are commonly used to delineate slick extent because of their day/night, all-weather imaging capability, their usefulness for directing response is severely limited because acquisitions only occur during satellite overpasses of the spill site; therefore, the instruments cannot be used to track short-term changes in the slick location, extent and oil characteristics, all of which can change rapidly in open water. Most satellite SARs in operation today do not regularly collect data over open waters except for specific areas of scientific and/or national interest, areas for which data are purchased and after the International Charter, Space and Major Disasters, is activated [13]. Rescheduling required to obtain images outside the operational plan can increase latency.

Today’s major challenges to effective spill response, remediation and restitution are accurate oil thickness measurement, short-term forecasting of slick transport, and estimation of release rates and volumes. To be of value during a response, the data needed to generate this information must be rapidly acquired and processed and the derived information distributed rapidly to spill responders. The methodologies used must be based on demonstrated and validated observations. Here, we show how these issues can be addressed with a suitable airborne SAR instrument and discuss the design and capabilities of an airborne SAR system that addresses at least in part each of these needs. We draw from data acquired by NASA’s Uninhabited Aerial Vehicle Synthetic Aperture Radar (UAVSAR) over recurring oil slicks in the Gulf of Mexico and Deepwater Horizon oil transported to coastal Louisiana (USA) to demonstrate airborne SAR’s potential for determining slick extent, tracking slick movement and characterizing thickness and emulsification in open or coastal waters. Finally, we discuss the information gap between what has been demonstrated to date and what is needed by responders and suggest areas of future research and development.

## 2. Instrument and Methods: Airborne SAR for Oil Spill Response and Recovery

### 2.1. Design and Response Capabilities of Airborne SAR

Oil slicks show up in SAR images from all platforms as relatively dark areas, often contrasting with the radar signature of surrounding water [14,15], and can be observed with X-band, C-band or L-band instruments operating in either horizontal (H) or vertical (V) polarization. The backscattered signal amplitude is reduced by oil primarily through damping of the small-scale ocean surface roughness and has been shown to agree well, from both satellite and aircraft platforms [16,17,18,19,20], with the tilted Bragg scattering k_B_ model [21]. In the Bragg model, the measured returns are the coherent backscatter from the ocean wave spectral component of the same wavenumber as the radar wave projected on the ocean surface, k_B_ = 2k_R_sin(φ_i_), where k_R_ is the radar wavenumber and φ_i_ is the incidence angle. For incidence angles between ~20 and 70 degrees, the highest return power is given by the vertical polarization returns (VV, or transmit V, receive V), followed by HH, then the cross-polarization signal, HV, which is generally substantially lower (~6 dB) than the co-polarized returns [22]. Satellite-borne radars have been used for decades in operational detection of slicks [12,23].

Compared to satellite SAR, airborne SAR has important advantages for urgent response, which include targeted deployment to a specific area, rapidity of image acquisition, short time repeat imaging capability and the potential of having a much higher signal-to-noise ratio (SNR) radar instrument. Because of the importance of rapid deployment, the most useful instruments are deployed on mobile platforms that can be targeted to specific areas without substantial delay, and aircraft have the advantage that they can travel long distances relatively quickly. However, as drones become more common and capable of carrying more volume and mass, they could provide the same advantages of aircraft, possibly at lower cost.

The primary key capability provided by airborne SAR relative to satellite instruments is rapid repeat imaging for slick tracking. Slick position vs. time can be used to initiate models that then forecast where the oil will be transported. This capability has been demonstrated with UAVSAR in a controlled oil release experiment in the North Sea [24]. In that experiment, a comparison of an oil slick transport model to a series of SAR images acquired at ~30-min intervals and local current measurements showed that knowledge of the local currents near the slick significantly improved the forecasted slick transport relative to using currents estimated from an operational ocean wave model alone. Local currents were measured through the release of a self-locating datum marker drift buoy (SLDMB) [25] within the slick, and this was used to estimate the near-surface currents by subtracting the wave-induced Stokes drift component from the SLDMB trajectory. 

The advantage of high SNR cannot be overstated. If the SNR is too low, the radar return power from an oil slick will be below the instrument noise floor, in which case, the signal can be used for the detection of slick extent, but not for differentiating zones of more or less oil within the slick [20]. Most satellite SAR instruments have little or no margin between the oil slick return and their noise floor [20,26,27], which seriously contaminates the returns and makes the instruments insensitive to oil characteristics. Observations indicate that the slick thickness is related to the damping of the capillary and gravity-capillary waves, with thicker layers causing more damping [16,28], i.e., less return power and darker images. Using imagery collected during the Deepwater Horizon oil spill in 2010, researchers have made advances in determining the volumetric oil fraction [16] and estimates of layer thickness [29,30] from SAR. Accurate information about thickness or volume is useful even in the absence of low latency product generation because it provides scientifically-supportable release volumes for remediation or restitution, information that is needed after clean-up has ended.

High spatial resolution is needed to identify small slicks, delineate slicks in near-shore areas and locate zones of thicker oil within slicks. Spatial averaging of high resolution single-look pixels is also an effective way to reduce noise and identify areas showing higher damping within slicks. The spatial resolution of a SAR instrument is primarily a function of the instrument bandwidth, which can be the same for an airborne or spaceborne instrument. In practice, spaceborne SARs have been limited by a combination of power requirements, on-board data storage and data downlink capacity, and tradeoffs are made between temporal sampling, swath coverage, multi-vs-single polarization and spatial resolution to fit within the system’s capabilities. On-board data storage is not a limitation for airborne SARs, so data can be nominally acquired at finer resolution with full swath capability and even with full polarization diversity.

Finally, airborne SAR needs the capability of rapid product generation and delivery to be useful for response. On-board processing can produce low resolution images that can be georeferenced, then downlinked as GIS-ready files or simple image files to responders in the field.

### 2.2. UAVSAR: A Testbed for Oil Spill Response

NASA first began to study oil slick characterization using airborne L-band SAR during the 2010 Deepwater Horizon oil spill in the Gulf of Mexico when images were acquired both over the main slick and along the Gulf coast. Following the encouraging results showing the sensitivity of SAR backscatter to the dielectric properties of the oil [16], which is related to the volumetric fraction of oil in the surface layer, a controlled release experiment in the North Sea was undertaken [31] in which weathering and transport of thin films (<1 μm) were studied. In 2016, the work continued in the Gulf of Mexico at the site of a persistent seep off the coast of Louisiana to study transport and weathering of a complex slick with thickness ranging from thin sheen through thick emulsions under natural conditions. All of the work was done using the UAVSAR platform, which is a NASA airborne science instrument.

**Figure 1:**
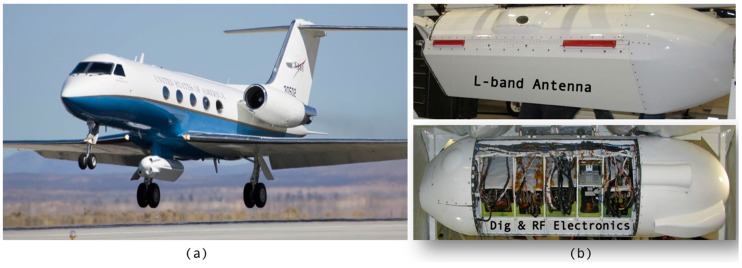
(**a**) UAVSAR platform with instrument pod located below the fuselage. (**b**) UAVSAR instrument.

**Figure 2:**
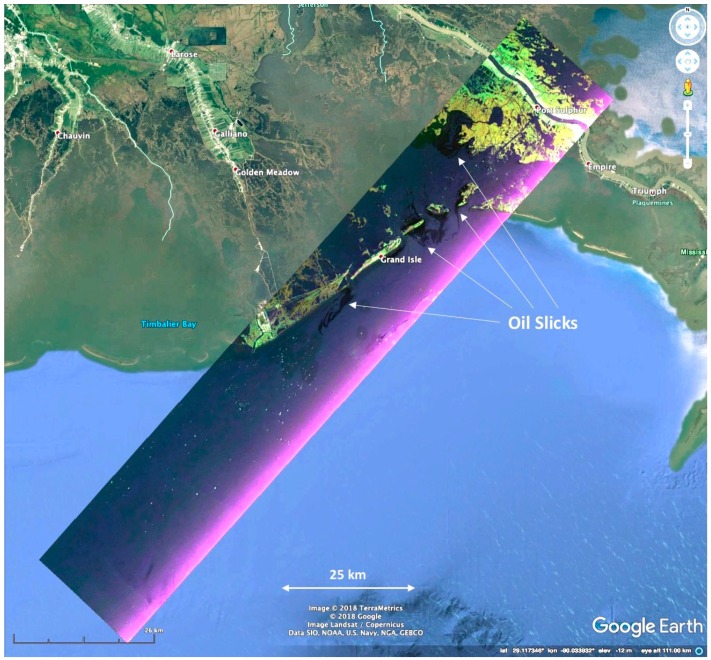
UAVSAR scene showing oil from the Deepwater Horizon spill along the Louisiana coast on 23 June 2010. The image swath is 132 km × 22 km. The colors are a composite of the HH-polarization intensity image (red), the VV (blue) and the HV (green). The oil slick is radar-dark in all polarizations. The near range is along the southern edge of the image and is brighter because the backscattered power is higher at smaller incidence angles (data source: UAVSAR scene ID Lamrsh_04201_10054, acquired 23 June 2010).

UAVSAR operates in the L-band (1.2575-GHz center frequency) with an 80-MHz bandwidth, which provides 1.7-m resolution in the slant range direction and has 0.8-m resolution in the along-track direction [32]. Typical products are generated with averaging to ~7-m spatial resolution. The radar operates in fully-polarimetric mode, meaning that it sequentially transmits vertical and horizontal polarization pulses and receives returns from each in both polarizations. The instrument is deployed on a Gulfstream-3 aircraft within a pod that mounts below the fuselage near the center of the aircraft (Figure 1). The antenna is flush mounted to the left side of the pod (left-looking), and the instrument images a swath ~22 km wide, with an incidence angle ~22° (near range) to ~67° (far range) in standard SAR side-looking geometry [30]. Typical flight operation is at a 12.5-km altitude (41,000’). The signal-to-noise ratio varies across the swath and is lowest at the edges and maximum near the swath center. Information on the instrument noise figure is provided in [33]. UAVSAR radar system characteristics are summarized in Table 1. Figure 2 shows a typical UAVSAR flight line of a swath width of 22.2-km overlaid on Google Earth. The scene shown was imaged in 10 min. Flight duration ranges from 5–6 h depending on weather conditions and altitude.

UAVSAR flight plans are developed prior to deployment and submitted to the U.S. Federal Aviation Administration (FAA) at least 24 h before radar operation. This is a strict requirement for radiating because UAVSAR operates at the same frequency as some of the commercial air traffic control (ATC) radars, and ATC centers are notified in advance that UAVSAR will be operating in their area. All flight lines (imaged swaths) potentially to be acquired must be in the plan, so re-planning the location of flight lines on-board can only involve selection between a fixed set of pre-defined lines that have been submitted to the FAA. For certain missions, in particular those involving imaging of ocean features, the exact location that should be imaged is not necessarily known in advance. For those studies, it is critical that there be some on-board processing capability so that quick-look views can be generated to determine whether the feature is imaged in a specific flight line.

**Figure 3:**
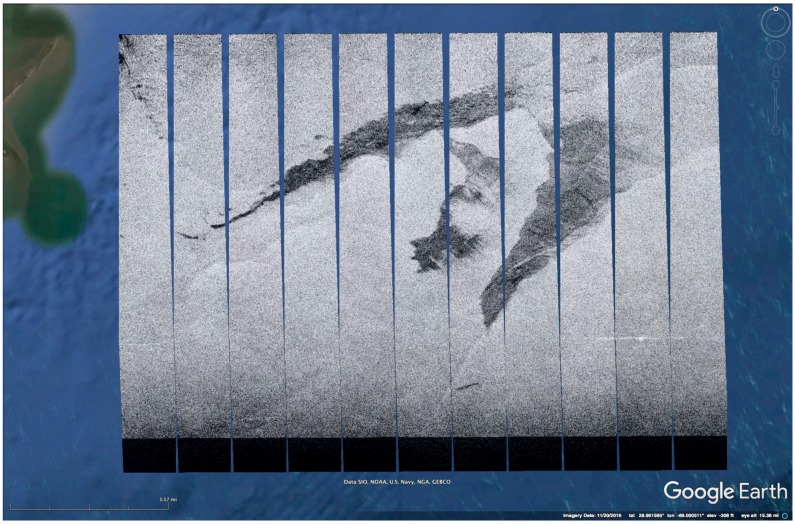
UAVSAR on-board processor (OBP) image showing a slick in the Gulf of Mexico, obtained 17 November 2016. The individual segments are processed and delivered over Ethernet to the operator on-board as the data are acquired. There is a ~1-min lag between when backscattered pulses are received and when the segment is delivered to the operator as a kml file. A simple script is used to automatically detect, download and display each segment as it is generated.

**Figure 4:**
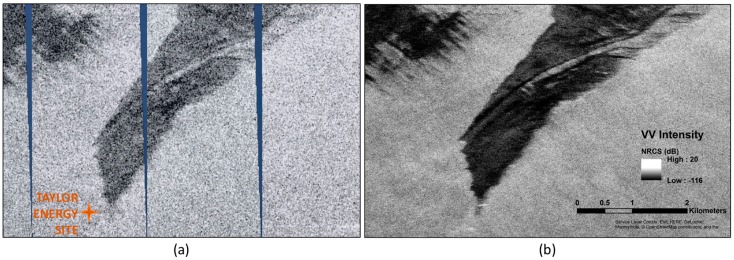
(**a**) OBP image compared to (**b**) the fully-processed intensity image showing calibrated values of the normalized radar cross-section (NRCS). The image shown is a subsection of the full OBP image in Figure 3. The location of the abandoned and destroyed Taylor Energy rig (no longer on the surface) is indicated in (**a**). Although the image contrast is clearly improved after full processing at higher resolution, contrast within the slick is apparent in the on-board product with sufficient information to direct responders to some areas of thicker oil (darker in image) (data source: UAVSAR scene ID gulfco_27086_16100, acquired 17 November 2016).

An on-board processor (OBP) unit has been developed for UAVSAR and is installed inside the cabin on the aircraft when needed for specific experiments. OBP images are generated as Google Earth files (kml) with low resolution and latency of approximately one minute between when the radar images the surface and when the kml file is produced. The images are provided in segments whose width is set by the processing algorithm, and are generated as the radar returns are acquired. Figure 3 shows the Google Earth screen with a series of 11 UAVSAR image segments displayed. Figure 4 shows a close-up OBP image of part of the slick, compared to the fully-processed image to show that some of the darker areas within the slick are visible in the near-real-time product. Higher resolution could be achieved on-board with longer processing time, more computing power and more storage. UAVSAR’s on-board processing capability includes file downlink to the ground, and other image formats could be accommodated with minor modification to the system. Downlink through a satellite phone has been demonstrated in the past, but the capability is not currently available on the aircraft.

## 3. Examples of Airborne SAR Spill Response Capabilities

Below, we draw from the results of the UAVSAR oil slick characterization science campaigns to show how this kind of instrument could be used for spill response, in particular in ways that distinguish it from satellite SARs. None of the work was done to provide information during a real urgent response event.

### 3.1. Oil Slick Tracking

Oil slicks can evolve quickly on the sea surface through transport both horizontally on the surface and vertically within the water column, and through weathering, which can cause loss of material through evaporation or concentration of material into emulsions that can persist for long times in the environment [9,34]. Accurately forecasting how a slick will evolve is difficult. Airborne SAR can image slick position directly without the need for daytime operation, e.g., the instrument could be used to determine the location of the slick before dawn to direct ground asset deployment or to image slicks in the Arctic during polar night. 

**Figure 5:**
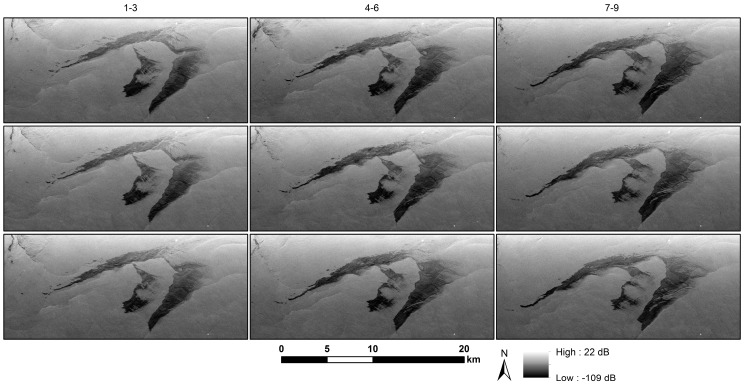
Transport and evolution of an oil slick in the Gulf of Mexico originating near the Taylor Energy rig site (see the rig location in Figure 4). The earliest image is at the upper left and the latest at the lower right, with Images 1–3 (4–6, 7–9) shown in Column 1 (2, 3). The images were acquired with 20 ± 1 min of separation. The general shape of the slick shows minor variation, but there are significant changes in backscatter intensity across the ~3.0-h interval between the first and last image. The Mississippi River outlet is just out of scene at the upper left. The slick spread initially to the northeast and then became entrained in the coastal along-shore current moving westward. The slick was also influenced by the spreading river plume to the northwest (middle-to-upper left in the images).

The oil slick tracking capability and its usage in the initiation and constraint of an oil transport forecasting model have been previously demonstrated using UAVSAR images acquired over intentional releases in the North Sea [24]. Here, we show another example of slick tracking drawn from a larger slick in the Gulf of Mexico with more internal variation, which was imaged by UAVSAR in November 2016. Figure 5 shows the evolution of the slick shown in Figure 3, in nine images collected at 20-min intervals over a 3-h time period. 

**Figure 6:**
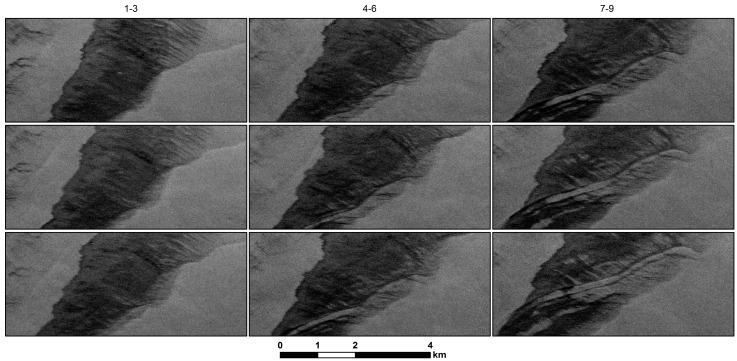
Zoom in of Figure 5 to show the development and propagation of an internal wave visible within the oil slick. Image order is the same as in Figure 5. In the later images (7–9, right-hand column), the internal wave is seen propagating northward at 0.5–1 km/h. Darker narrow bands, likely thicker concentrations of oil, collect along two convergence zones of the leading and trailing edges of the internal wave.

Figure 6 shows a close-up view of an area within the slick where an internal wave appeared to develop during the last half of the acquisitions. This example shows how quickly changes occur within the slick, how fast the slick can move and hence how difficult it would be for a boat to track and follow the changes from a sea-level reference frame only. As can be seen, the internal wave propagates northward. The two dark narrow bands on each side of the wave likely contain oil that has accumulated preferentially in the convergence zones of near-surface internal waves [35]. It is not clear that other, shorter wavelength components of an internal wave packet are detectable in this sequence. As pointed out for Figure 5, the presence of the river plume and the along-shore current impacts the spreading of the oil slick and provides complicated ocean conditions for internal wave generation along with local tides and local currents. For this study, we point to the evolving appearance of this feature to illustrate radar backscatter variations of a slick over a short temporal interval and the complexity and rapid variability of slick transport in near-shore locations.

### 3.2. Oil Slick Characterization

Although in the past, it has been thought that SAR had limited value for spill response, useful only for delineating slick extent [10,12], there is evidence that a suitably low noise instrument can detect emulsions [16], differentiate thick from thin oil [29,30] and quantify oil:water volumetric fraction [16]. A large number of parameters has been suggested as the best for detecting or characterizing oil slicks, but recent results using low noise airborne SARs have shown that the most reliable parameters are the VV intensity; the VV damping ratio, i.e., the contrast between the VV-intensity in clean vs. slicked water (VV^clean^/VV); and the polarization difference (VV-HH) [17,18].

For operational airborne SAR-based slick characterization, the most practical of the parameters is the VV damping ratio because only one polarization mode is needed, significantly reducing the instrument complexity. The ratio also reduces dependence on the incidence angle and, for the case shown, the incidence angle dependence of the intensity, pointed out earlier in Figure 2. For the slick in Figure 5, the incidence-angle dependence largely cancels in the damping ratio across the part of the scene with the slick. The VV damping ratio has been used for decades now as a reliable parameter for determining slick extent [19].

**Figure 7:**
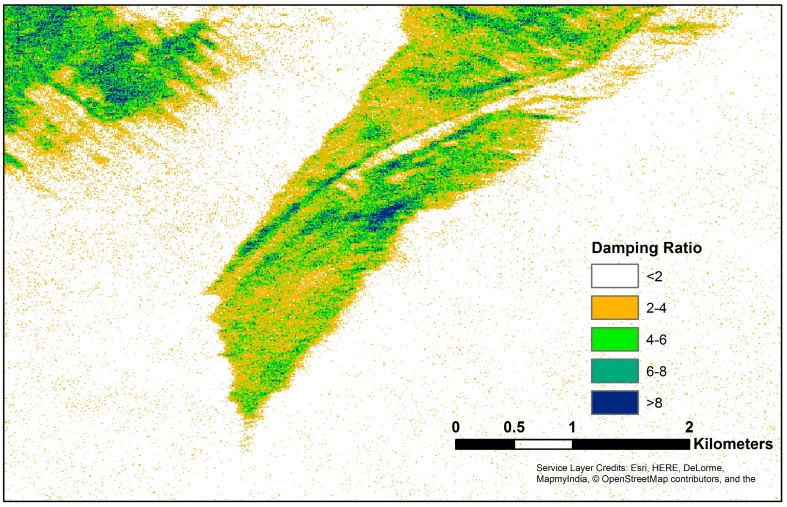
VV damping ratio for the slick shown in Figure 4. Higher damping ratios are apparent in the narrow convergence zones on both sides of the internal wave and at several locations within the slick.

Figure 7 shows the VV damping ratio for the slick shown in Figure 4. For the damping ratio, the clean sea values were chosen visually by selecting large areas without apparent boats, fronts or slicks, determining VV^clean^ as a function of incidence angle and taking the ratio for the full scene accounting for the incidence angle of each pixel. The slick shows up well with damping ratios >2, a threshold for which there are scattered false positive points within the clean water. We note that this threshold depends on the sea state and meteorological conditions, primarily the wind field, and more study is needed to quantify the relationship. It is not known whether the damping is due to thicker layers of fresh oil or weathered oil-water emulsions (themselves usually thicker [10]); however, the clean-up response would benefit from knowing either of those two oil conditions.

**Figure 8:**
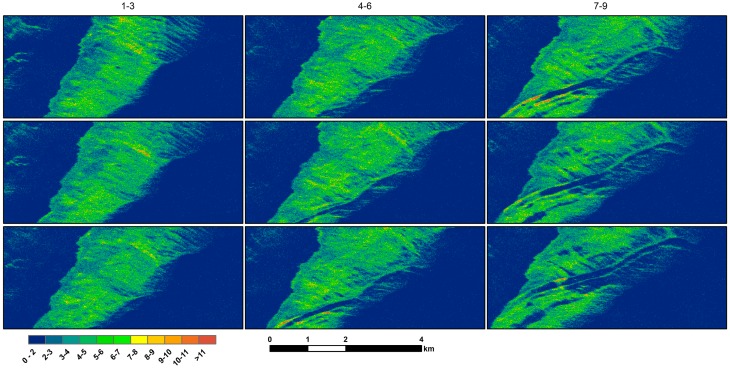
VV damping ratio for the series of images shown in Figure 6. Image order is the same as in Figure 5. Higher damping is seen in persistent features that remain on the surface in many instances for more than 20 min (the interval between individual scenes).

Figure 8 shows how the damping ratio changes with time, as an example of combining airborne SAR’s tracking and characterization capabilities. In this case, some areas of higher damping are observed to be transported with the slick or converge and move along the front and trailing edges of the internal wave. This series of plots shows how rapidly the areas of thicker/more emulsified oil can move and change. 

### 3.3. Slick Identification in Nearshore Waters

**Figure 9:**
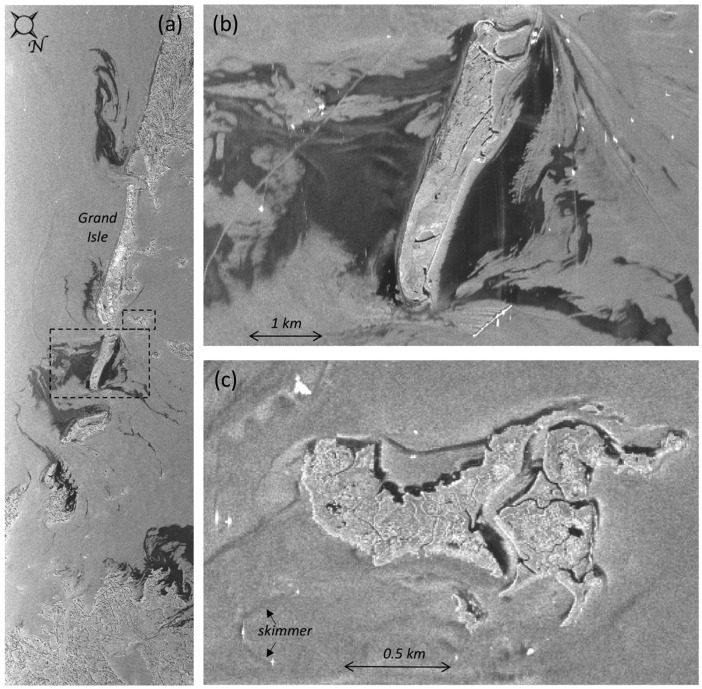
The VV intensity images of (**a**) slicked waters along the Louisiana coastline (from Figure 2) with boxes showing the locations of sub-scenes to the right; (**b**) Isle Grand Terre; (**c**) Beauregard Island. The waters of the Gulf of Mexico are to the left of the barrier islands shown. The UAVSAR imagery was obtained on 23 June 2010.

The problem of unambiguously identifying and tracking slicks in coastal and inland waters is likewise challenging as it is in open water, but with additional complications. Firstly, higher resolution imagery is needed to resolve slicks near the shore. Secondly, near-coastal waters are generally calmer, thus displaying lower radar backscatter even without slicks present. This is particularly true on the lee side of islands. Furthermore, biogenic slicks are often present in coastal areas and have low backscatter as well. At this point in time, there is no reliable way to differentiate false positive dark returns from oil slicks using SAR. An example of the complexity of the situation is shown in Figure 9, which is a close-up of the VV intensity of the scene shown in Figure 2, focusing on slicks near the Louisiana coastline. To retain full resolution, the images displayed in Figure 9 are not georeferenced, but displayed in radar coordinates with the incidence angle increasing from left to right. We focus on the slicks near Isle Grand Terre, the island below (in the image) Grand Isle, Louisiana, and Beauregard Island, a small island inland from the north end of Grand Isle. The larger areas of radar-dark areas in the Gulf of Mexico are likely slicks from the Deepwater Horizon spill, and the variation in image intensity within the slick on the ocean (left) side of the island seen in Figure 9b supports the conclusion that this is an oil slick. It is more difficult to determine whether the dark area on the leeward (right) side of Isle Grand Terre (Figure 9b) is a mineral oil slick or calm winds or both. Firstly, the intensity is lower than that on the opposite side of the island because the water is calmer. The features supporting that these areas are oil include (1) the radar-dark area extends further offshore than would be expected for leeward calm waters alone and (2) smaller slicks extend from this island as long, thin features, although the latter is also a characteristic feature of biogenic slicks. This contrasts with the radar-dark areas around Beauregard Island, shown in Figure 9c. In this case, the dark areas closely follow the coast and even show up in channels within the island. Based on intensity alone, we cannot tell whether these are oil slicks or not, and information on local winds and tidal flow would aid the determination. However, we can tell where there are not slicks by the brighter return, clean water. We note that the skimmer (bottom of Figure 9c) would have benefited from guidance based on this image. 

We point out that several current satellite SAR instruments (for example, TerraSAR-X, Radarsat2) have selectable imaging modes with resolutions comparable to that of UAVSAR, but they have significantly lower signal-to-noise ratios. We return to the unique utility of a time series of high resolution images obtainable via aircraft that could help determine whether areas of low backscatter are oil slicks or calm winds, which can evolve differently even over a short time interval [36].

## 4. Discussion

Airborne SAR offers distinct and substantial advantages over satellite SARs for response to oil spills in flexibility of time and location of imaging. For the several-hour time series shown in Figure 5, only one coincident image by a single satellite SAR would have been possible, but a single image by itself would not be sufficient to track slick transport or its evolving properties. Other advantages include the following: higher SNR, which can substantially improve tracking of the transport and evolution of an oil slick and characterizing the zones of thicker or more emulsified oil within a slick; and combined high SNR and spatial resolution for identifying slicks in water very near or in contact with land. Examples from studies undertaken with the NASA UAVSAR airborne L-band SAR show the potential of the airborne instruments for each of these [16,17,18,20,24,26,29]. We have also shown examples of UAVSAR’s on-board SAR processing, which would be a key component of a response platform. A validated oil detection and thickness algorithm could be implemented on an aircraft and incorporated into an on-board processor. This would enable more efficient transmission of analyzed imagery to a response center rather than downloading the much larger raw data files, which would still require processing to obtain an oil spill characterization product.

There are also disadvantages, with which we have direct experience in our studies: as mentioned previously, UAVSAR is a science instrument, not an urgent response platform, so it has only been used for oil spill research. The oil spill response community would need to develop, maintain and deploy an airborne SAR instrument or instruments for response. In this case, a relatively large aircraft with long-range capability or a fleet of smaller aircraft would be needed to cover, e.g., the continental United States and Alaska. Given that the response community does not pay for the satellite SAR missions, an airborne SAR system and platform will be more expensive than a satellite SAR system, albeit with the clear advantage of timely and targeted imaging capability. Secondly, airborne SARs for this application need to be high power, and care needs to be taken in their design to maintain a margin of >6 dB above the noise floor [16,17,18]. Studies of returns from slicks under the expected range of wind conditions and sea states are needed to determine the necessary SNR for a given region. Thirdly, in very low wind conditions (<~1.5–2 m/s, based on experience with UAVSAR), slicks cannot be distinguished from clean water, and in white-capped water (high wind, >~14 m/s), returns from slicks are bright like the returns from un-slicked water, reducing the range of possible environmental conditions in which oil slicks can be observed with SAR.

There are a number of challenges remaining for SAR to provide the quantitative information that would be optimal for oil spill response or recovery operations. Although oil volumetric fraction estimation has been done based on SAR for very thick slicks [16], more work needs to be done to validate the results. Regarding oil layer thickness or volume, we have only shown here relative thickness determination at a level that is not equivalent to the Bonn slick classification. Validation against field measurements needs to be done. An area of open research is to quantify oil thickness, using either a physics-based model or an empirical model. More research in this area needs to done and will require a better and more detailed understanding of how sea state and wind speed alters an oil layer’s damping of the ocean wave spectra. This is an area we are currently pursuing, with a focus on optimizing the use of an instrument like UAVSAR and providing guidance on the design and development of an operational SAR aircraft system for oil spill response.

## Figures and Tables

**Table 1:** Uninhabited Aerial Vehicle SAR (UAVSAR) system characteristics.

Parameter	Value
Frequency	1.2575 GHz
Wavelength	23.79 cm
Bandwidth	80 MHz
Polarization	HH, HV, VV, VH
Operating Altitude	12.5 km (typical)
Ground Speed	220 m/s (typical)
Swath Width	22 km
Slant Range Resolution	1.7 m
Along Track Resolution	0.8 m
Transmit Power	3 kW
Noise Equivalent Sigma Naught	>−55 dB (see [33] for the profile)
